# Dietary cystine restriction increases the proliferative capacity of the small intestine of mice

**DOI:** 10.1371/journal.pone.0290493

**Published:** 2024-01-05

**Authors:** Judith C. W. de Jong, Kristel S. van Rooijen, Edwin C. A. Stigter, M. Can Gülersönmez, Marcel R. de Zoete, Janetta Top, Matthijs J. D. Baars, Yvonne Vercoulen, Folkert Kuipers, Saskia W. C. van Mil, Noortje Ijssennagger

**Affiliations:** 1 Center for Molecular Medicine, University Medical Center Utrecht, Utrecht, The Netherlands; 2 Department of Medical Microbiology, University Medical Center Utrecht, Utrecht, The Netherlands; 3 Department of Pediatrics and Laboratory Medicine and European Research Institute for the Biology of Ageing (ERIBA), University of Groningen, University Medical Center Groningen, Groningen, The Netherlands; University of Hawai’i at Manoa, UNITED STATES

## Abstract

Currently, over 88 million people are estimated to have adopted a vegan or vegetarian diet. Cysteine is a semi-essential amino acid, which availability is largely dependent on dietary intake of meat, eggs and whole grains. Vegan/vegetarian diets are therefore inherently low in cysteine. Sufficient uptake of cysteine is crucial, as it serves as substrate for protein synthesis and can be converted to taurine and glutathione. We found earlier that intermolecular cystine bridges are essential for the barrier function of the intestinal mucus layer. Therefore, we now investigate the effect of low dietary cystine on the intestine. Mice (8/group) received a high fat diet with a normal or low cystine concentration for 2 weeks. We observed no changes in plasma methionine, cysteine, taurine or glutathione levels or bile acid conjugation after 2 weeks of low cystine feeding. In the colon, dietary cystine restriction results in an increase in goblet cell numbers, and a borderline significant increase mucus layer thickness. Gut microbiome composition and expression of stem cell markers did not change on the low cystine diet. Remarkably, stem cell markers, as well as the proliferation marker *Ki67*, were increased upon cystine restriction in the small intestine. In line with this, gene set enrichment analysis indicated enrichment of Wnt signaling in the small intestine of mice on the low cystine diet, indicative of increased epithelial proliferation. In conclusion, 2 weeks of cystine restriction did not result in apparent systemic effects, but the low cystine diet increased the proliferative capacity specifically of the small intestine and induced the number of goblet cells in the colon.

## Introduction

Cysteine is a semi-essential amino acid, which is provided by dietary intake of meat, eggs and whole grains. Cysteine can also be synthesized from the essential amino acid methionine via the transsulfuration pathway [1, 2]. The majority of dietary cysteine is absorbed in the small intestine. Its ileal uptake occurs by the cystine/glutamate exchange transporter, xCT [3]. Unabsorbed cysteine travels to the colon where it can be converted by sulfate- or sulfite- reducing bacteria to hydrogen sulfide. Cystine is the oxidized form of cysteine. Compared to cysteine, cystine from food sources is absorbed less efficiently from the small intestine due to its lower digestibility [4]. Intracellularly, cystine is reduced to cysteine by the NADH-dependent enzyme cystine reductase [5].

Besides being used for protein synthesis, cysteine can be converted to taurine, glutathione and hydrogen sulfide in the body. These three metabolites have differential effects on intestinal function. Taurine is important for bile acid conjugation in the liver. In humans, bile acids can be conjugated with either glycine or taurine (3:1), while mice conjugate 95% of bile acids with taurine [6]. Specific taurine-conjugated bile acids like taurocholic acid and taurolithocholic acid have been described to increase proliferation in intestinal cell lines [7, 8]. Additionally, taurine-conjugated bile acids increase the abundance of the sulfite-reducing bacteria B. Wadsworthia, which results in an increase in hydrogen sulfide production [9].

Hydrogen sulfide maintains the integrity of the mucus layer when derived from endogenous metabolism, but is detrimental when produced in high concentrations by the gut microbiome [10]. We previously showed that hydrogen sulfide breaks disulfide bonds in mucus, thereby opening the mucus barrier [11, 12]. This reduces the protective capacity of the mucus barrier and increases the exposure of epithelial cells to toxic compounds present in the lumen.

Lastly, glutathione (GSH) is an antioxidant which protects the intestine from oxidative stress and DNA damage. The addition of glutathione to intestinal porcine enterocytes on a cysteine-deprived medium restores proliferation and cell viability by replenishing the cysteine pool [13]. Most of the above mentioned studies mimick high cystine/cysteine intake, with concurrent high concentrations of GSH, taurine and hydrogen sulfide, equivalent to a diet high in animal protein intake. Previously, we [11] and others [14] showed that mice on a low cystine diet can conjugate their bile acids with glycine instead of taurine, which might affect intestinal characteristics. As there is a trend towards adopting vegetarian and vegan diets, which are low in cystine [15], we investigate the effect of a low cystine diet on intestinal function in this study. We show that the low cystine diet did not affect the colon, except for an increase in the number of goblet cells. In the small intestine however, cystine restriction results in increased epithelial proliferation and in an increase in the number of stem cells associated with increased Wnt signaling.

## Results

### Dietary cystine restriction does not affect metabolism into cystine breakdown products

**Fig 1:**
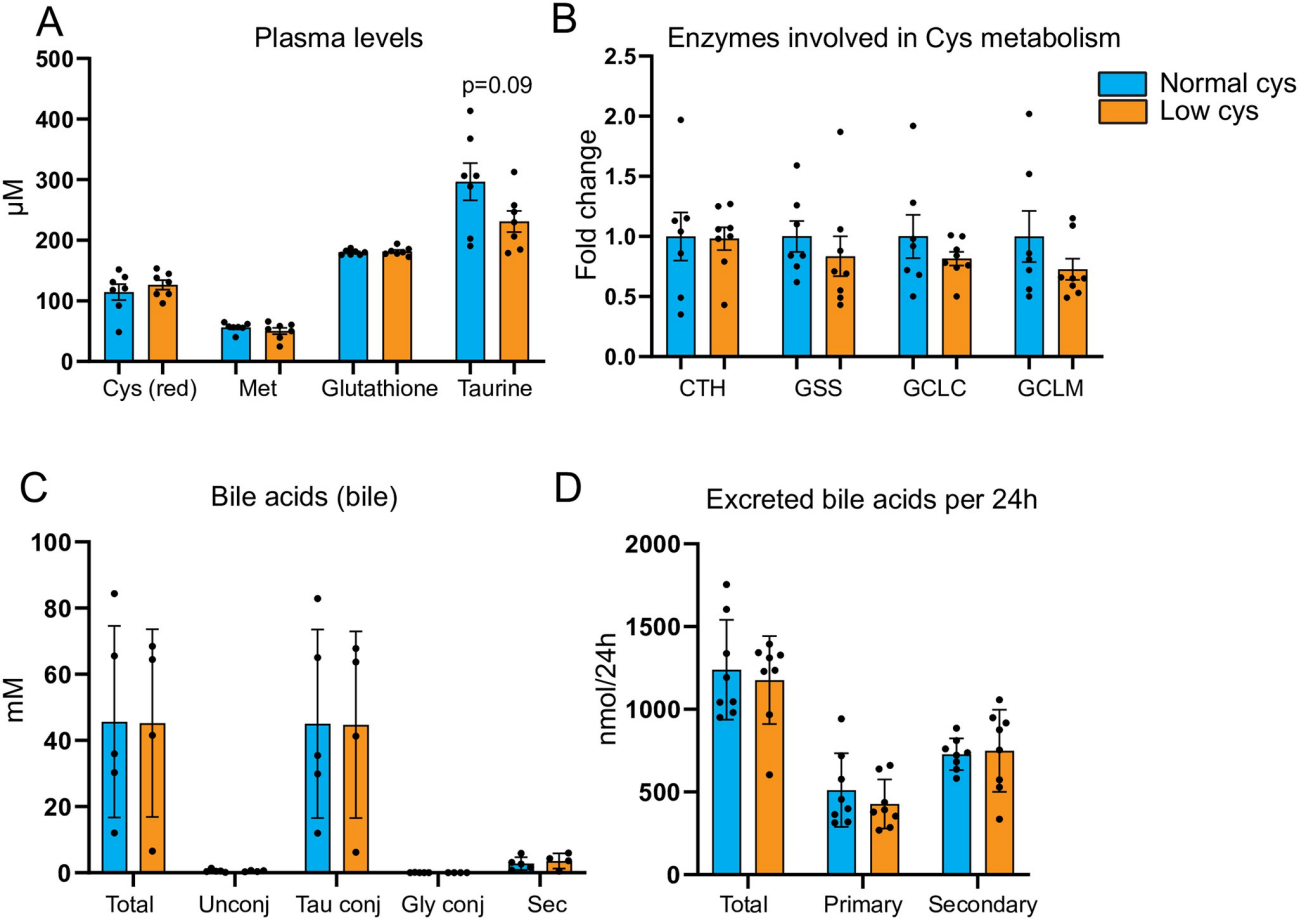
Dietary cystine restriction does not affect metabolism into cystine breakdown products. A) Plasma cysteine (reduced) (abbreviated as Cys (red)), methionine (Met), glutathione and taurine levels measured by metabolomics (n = 8/group, mean ± SEM). B) qPCR gene expression of enzymes converting methionine to cysteine (CTH) and cysteine to glutathione (GSS, GCLC, GCLM) normalized by normal cys (n = 8/group, mean ± SEM). C) Bile acid concentrations measured in bile (n = 5/normal cys; n = 4/low cys, mean ± SEM). D) Excreted bile acids per 24h (right), measured in feces (n = 8/group, mean ± SEM).

Mice (n = 8/group) received either a high fat (40 en%) diet or a high fat diet low in cystine (low cys) for two weeks. After 2 weeks of intervention, there was no difference in the body weight of these mice (S1A Fig). The cystine-restricted diet did not cause a reduction in plasma cysteine levels (Fig 1A), suggesting that either the dietary deficiency is compensated for by methionine-to-cysteine conversion, or by a reduced production of taurine, glutathione and/or hydrogen sulfide. Plasma methionine levels were also not changed (Fig 1A), neither was the mRNA expression of cystathionine γ-lyase (*CTH*) (Fig 1B), which is responsible for the last step in the conversion from methionine to cysteine. This suggests that the methionine-to-cysteine conversion is not increased. The cystine-restricted diet did not impact on glutathione production either, as both the plasma glutathione concentration itself and mRNA expression of enzymes involved in the conversion from cysteine to glutathione (*GSS*, *GCLC*, *GCLM)* were unchanged (Fig 1A and 1B). No significant changes in other plasma amino acids were observed (Table 1), suggesting that the 2-week cystine restriction consumption does not lead to any systemic effects. Cystine restriction resulted in slightly decreased plasma taurine concentrations (Fig 1A), however this was not significant. We previously observed that mice on a low cystine diet can conjugate bile acids with glycine instead of taurine [11]. Also, methionine-restriction has been shown to increase the ratio glycine to taurine conjugation of bile acids in mice [14]. Here, we do not find changes in the bile acid conjugation (Fig 1C), or excretion into feces (Fig 1D). Taken together, reduction to 0.08% cystine for two weeks did not affect the systemic availability of cysteine, its downstream catabolic metabolites, or bile acid conjugation.

**Table 1:** Concentrations of other plasma amino acids measured using metabolomics.

	Normal cys (average ± SD)	Low cys (average ± SD)
**Arginine (μM)**	88.5 ± 16.8	90.7 ± 11.2
**Glycine (μM)**	295.2 ± 31.7	261.1 ± 33.3
**Aspartic Acid (μM)**	12.6 ± 2.0	12.6 ± 2.4
**Citrulline (μM)**	56.7 ± 5.8	55.1 ± 10.0
**Glutamic Acid (μM)**	56.9 ± 10.6	58.2 ± 9.8
**Alanine (μM)**	422.7 ± 83.6	376.3 ± 120.6
**Tyrosine (μM)**	66.3 ± 13.4	62.0 ± 14.3
**Valine (μM)**	229.9 ± 28.9	237.2 ± 23.4
**Leucine (μM)**	152.0 ± 21.1	153.9 ± 23.3
**Phenylalanine (μM)**	61.3 ± 7.8	59.1 ± 6.6
**Histidine (μM)**	70.2 ± 7.7	70.1 ± 7.7
**Asparagine (μM)**	49.6 ± 7.0	44.4 ± 9.8
**Serine (μM)**	158.1 ± 23.7	143.8 ± 37.0
**Glutamine (μM)**	520.0 ± 66.9	460.1 ± 71.7
**Threonine (μM)**	90.3 ± 6.4	88.8 ± 9.1
**Proline (μM)**	91.1 ± 13.1	84.2 ± 17.3
**Ornithine (μM)**	46.6 ± 18.6	46.3 ± 5.3
**Lysine (μM)**	186.3 ± 33.7	184.1 ± 22.9
**Isoleucine (μM)**	100.3 ± 17.2	98.1 ± 6.9
**Tryptophan (μM)**	78.5 ± 8.9	75.1 ± 12.8

### Dietary cystine restriction increased the amount of goblet cells in the colon

**Fig 2:**
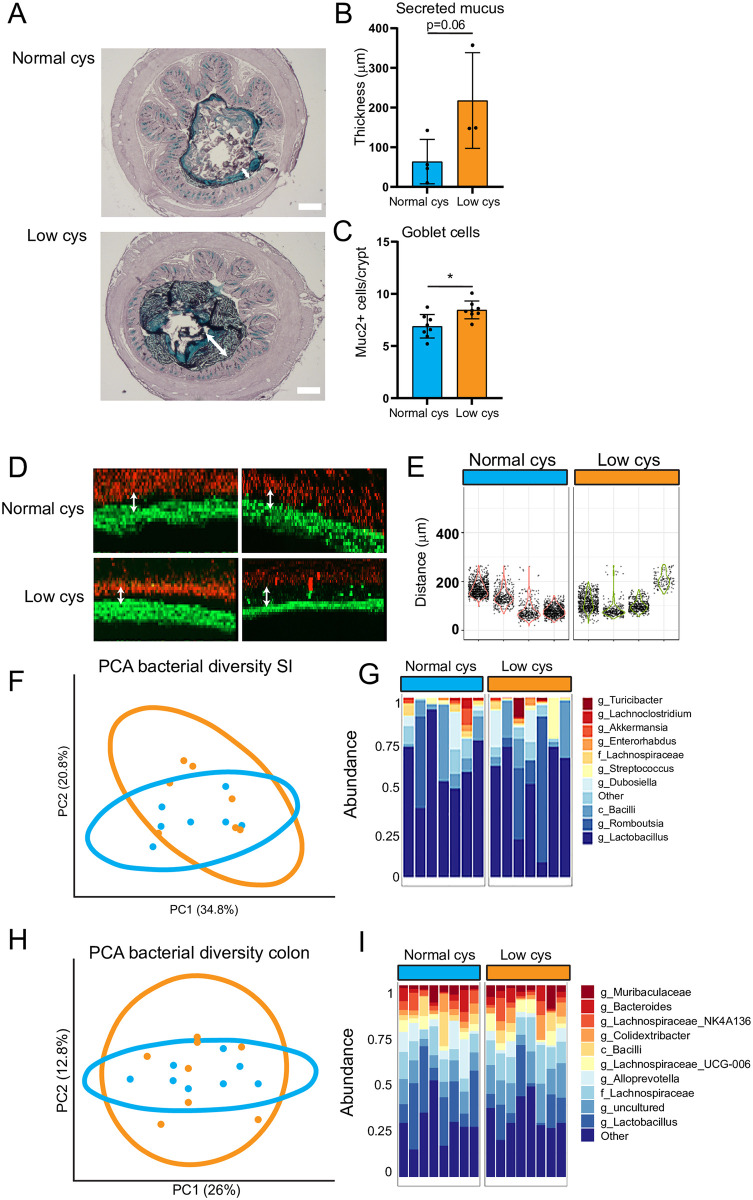
Dietary cystine restriction increased the amount of goblet cells in the colon. A) Representative images of the secreted mucus layer visualized by a High Iron Diamine (HID) staining (scalebar = 200 μm). Arrows indicate mucus layer thickness. B) Quantification of the secreted mucus layer (n = 4 for normal cys; n = 3 for low cys, average of 12 measurements per mouse ± SEM are depicted, Mann-Whitney test). C) Quantification of Muc2-positive goblet cells, per crypt (n = 8/group, mean ± SEM, unpaired t-test, *p<0.05). D) Permeability of the distal colonic mucus layer was visualized by adding 1-lm fluorescent beads (red) to the epithelial tissue (green). Shown are representative maximum projections of a resliced z-stack, showing a summarized view of the sample from the side. Arrows indicate distance. E) The distance between the beads and the epithelium was measured and depicted for 4 mice per group (Each dot represents one bead). F) PCA plot for the diversity of the small intestinal microbiome. Each dot represents one mouse (n = 7/group). G) Relative abundance of microbiota in the small intestinal contents at the g (genus), f (family), c (class) level in individual samples (mice were individually housed). H) PCA plot for the diversity of the colonic microbiome. Each dot represents one mouse (n = 8/group). I) Relative abundance of microbiota in the colonic contents at the g (genus), f (family), c (class) level in individual samples (mice were individually housed).

Cystine molecules escaping absorption in the small intestine, eventually reach the colon. The colonic epithelium is covered by mucus consisting of mucin polymers connected via disulfide bonds. This mucus layer limits the exposure of epithelial cells to toxins and bacteria. Certain bacteria such as sulfate reducing bacteria, reduce these disulfide bonds [11], breaking the mucus barrier and increasing the exposure of epithelial cells to toxins and bacteria present in the lumen. Cystine also contains disulfide bonds which can be used as substrate for bacteria. We hypothesized therefore that decreased luminal concentrations of cystine might increase the chance that bacteria reduce the disulfide bonds from mucus rather than cystine. We observed that dietary cystine restriction showed a borderline significant increase towards an increase in the thickness of the secreted mucus layer (Fig 2A and 2B). There was a significant increase in the number of goblet cells on the low cystine diet (Fig 2C). Mucus barrier capacity, as measured by fluorescent bead penetration *ex vivo*, was not altered (Fig 2D and 2E). Since the small intestine lacks an inner sterile mucus layer, there was no reason to study small intestinal mucus properties. No differences in bacterial composition upon dietary restriction of cystine were observed, neither in the small intestinal contents (Fig 2F and 2G), nor in the colonic contents (Fig 2H and 2I), indicating that the effects on the mucus barrier were not caused by changes in the abundance of mucus-degrading- or sulfate reducing bacteria.

### Dietary cystine restriction increases the expression of stem cell markers and proliferation specifically in the small intestine

**Fig 3:**
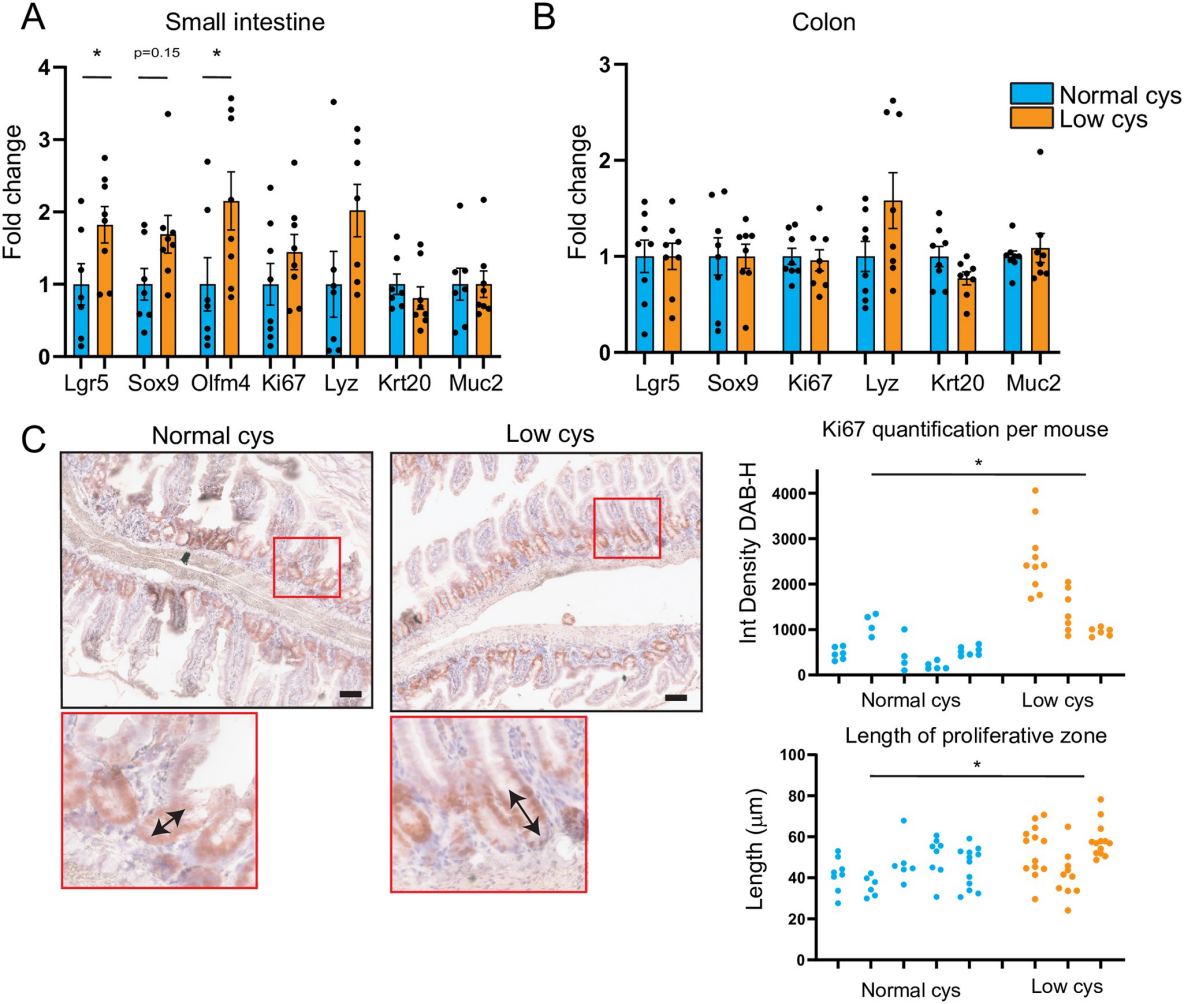
Dietary cystine restriction increases the expression of stem cell markers and proliferation specifically in small intestine. qRT-PCR gene expression in A) small intestine and B) colon of markers of intestinal stem cell (Lgr5, Sox9, Olfm4), proliferative cells (Ki67), enterocyte (Krt20), Paneth cell (Lyz) and goblet cells (Muc2) normalized for normal cys (n = 8/group, mean ± SEM, multiple Mann-Whitney tests, *p<0.05). C) (Left) Representative images of Ki67 immunohistochemistry on small intestinal crypts (scale bar = 50 μm). Arrows indicate length of proliferative zone. (Top Right) Quantification of integrated density of Ki67 corrected for haematoxylin, as measured using Fiji, of multiple crypts per mouse. Each column represents one mouse (n = 5 for normal cys, n = 3 for low cys, unpaired t-test, *p<0.05). (Bottom Right) Quantification of the length of the proliferative zone, using the length tool in Fiji, stained by Ki67 of multiple crypts per mouse. Each column represents one mouse (n = 5 for normal cys, n = 3 for low cys, unpaired t-test, *p<0.05).

Next, we investigated if other cell types besides goblet cells were changed upon cystine restriction. Gene expression levels of stem cell (*Lgr5*, *Sox9*, *Olfm4)*, proliferative cell (*Ki67*), Paneth cell (*Lyz)*, enterocyte (*Krt20)* and goblet cell (*Muc2)* markers were determined in both small intestine (Fig 3A) and colon (Fig 3B) using qRT-PCR. Stem cell markers (*Lgr5*, *Sox9*, *Olfm4)* were, or tended to be, significantly increased upon cystine restriction in the small intestine. Markers of other cell types were not affected. The increase in stem cell markers in the small intestine was accompanied by higher Ki67 mRNA and protein expression, indicative of increased epithelial proliferation (Fig 3C). In addition, the zone of Ki67-positive cells was elongated upon cystine restriction (Fig 3C). Remarkably, these effects on stem cell markers and on proliferation were specific for the small intestine, as in the colon no changes were observed (Fig 3B).

An increase in proliferation and stem cells can be indicative of intestinal damage and the necessity to replenish the damaged cells [16, 17]. However, since we did not observe any significant changes in colon crypt length (S1B Fig) nor in expression of intestinal damage markers (*Ier3*, *Ripk3*, *Birc5*, S1C and S1D Fig) [11], there were no indications of epithelial cell damage upon cystine restriction for 2 weeks.

### Cystine-restriction mediated increase in proliferation correlates with an increase in Wnt signaling

**Fig 4:**
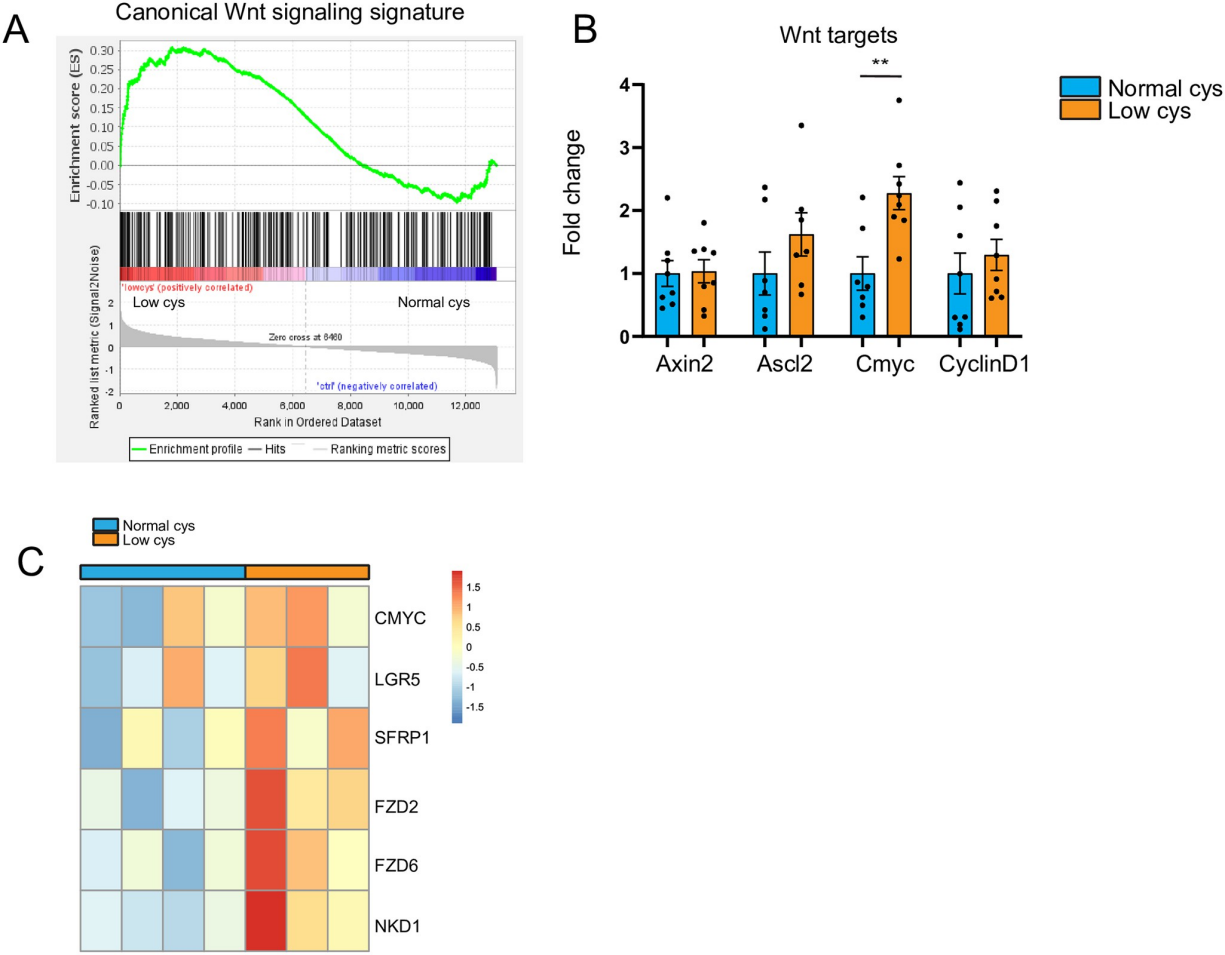
Dietary cystine restriction increases Wnt signaling in small intestine. A) Gene set enrichment analysis for a Wnt signaling signature reveals significant enrichment with low cys vs ctrl (NES = 1.37, FDR = 0.07). B) qPCR gene expression analysis of Wnt target genes (n = 8/group, mean ± SEM, multiple unpaired t-tests, **p<0.01). C) Heatmap of multiple enriched Wnt target or signature genes from the RNAseq data comparing low cys to normal cys (expressed as log fold change).

To investigate the cause of increased proliferation in the small intestine upon cystine restriction, we performed RNA sequencing analysis on the small intestine. Dietary cystine restriction only led to very subtle transcriptomic changes; only 16 genes were significantly differentially expressed when comparing low cystine to normal cystine (S2A Fig). RNA seq was also performed in the colon, but there we did not find significant different genes at all (data not shown). Most of differentially expressed genes in the small intestines were upregulated in the low cystine diet group and represent either pseudogenes or genes part of the immunoglobulin kappa variable cluster (IGKV). However, gene set enrichment analysis (GSEA) revealed that one of the major pathways involved in intestinal stem cell homeostasis; Wnt signaling, was significantly enriched (FDR = 0.07) upon a low cystine diet (Fig 4A). This was confirmed by qRT-PCR by the significant increase of the Wnt target gene *Cmyc* (Fig 4B) and by the increased expression of 6 Wnt target and signature genes in the RNAseq data (Fig 4C). As increased Wnt signaling is known to drive proliferation and is essential for stem cell maintenance, the increased Wnt signaling seen in cystine-restricted mice, most likely caused the observed effects on stem cells and proliferation.

## Discussion

Vegan and vegetarian diets contain limited amounts of cystine/cysteine, and although the human body can produce cysteine from methionine, the daily cysteine requirements are largely dependent upon direct dietary intake of cystine and/or cysteine. In this study, we therefore investigated the effect of dietary cystine restriction on the gut epithelium of mice. To our knowledge, this is the first study showing that cystine restriction affects epithelial proliferation and the expression of stem cell markers in the small intestine of mice, most probably involving increased Wnt signaling. There was no effect of cystine restriction on proliferation in the colon. In the colon, dietary cystine restriction led to an increase in goblet cells and a trend towards an increase in mucus layer thickness.

Cystine restriction did not result in changes in plasma levels of cysteine, methionine, taurine and glutathione, indicating that there were no systemic effects of the low cystine diet. No changes in the microbiota were observed either, implying that the observed effects are most likely caused by the difference in cystine levels in the luminal content. The limiting systemic effects in our study could be attributed to several factors, including a relatively short duration of the diet (14 days) or the fact that the low cystine diet still contains 0.08% of cystine, which might not be limiting enough. The required level of cystine in the AIN93G diet is 0.38%, of which 0.08% of cystine comes from casein and 0.3% of cystine is added. For our restricted diet, the normally added 0.3% of cystine was replaced equimolarly with alanine. Other studies report that 12-weeks feeding of a cystine restricted diet in rats and mice result in more pronounced effects, at least on plasma amino acids such as taurine [18, 19]. Moreover, even lower concentrations of cystine (0.06% and 0.03%) have been reported to decrease plasma methionine concentrations in rats [19]. The latter paper also suggests that a diet restrictive in both methionine and cystine already affects lipid metabolism related parameters within 2 weeks in mice. This suggests that cystine-restriction alone for 2 weeks is not sufficient to cause systemic effects in two weeks.

Certain bacteria such as sulfate reducing bacteria, reduce disulfide bonds present in colonic mucus [11], thereby breaking the mucus barrier and increasing the exposure of epithelial cells to toxins and bacteria present in the lumen. Cystine also contains disulfide bonds which specific bacteria use as substrate. Decreased luminal concentrations of cystine might therefore provide a shift in substrate usage by bacteria. We speculate that the observed trend towards an increase in the thickness of the secreted mucus layer is representing a more reduced or open outer mucus layer, because the reduced luminal cystine concentrations force bacteria to shift to mucus as a substrate for di-sulfide bond splitting. This hypothesis is further supported by a study reporting that cystine supplementation has a mucosal barrier enhancing effect [20]. Whether this mechanism plays a role in the current study needs to be further investigated, but the fact that there are more goblet cells in the colon in the low cystine group, even though the inner sterile layer is not thicker, might hint at a compensatory mechanism to produce more mucins.

This study shows that cystine restriction resulted in increased epithelial proliferation. In literature, contradictory effects of cysteine, often in combination with methionine, on intestinal proliferation have been reported in diverse disease states and animal models. Both sulfur amino acid (methionine and cysteine) restriction and supplementation diets have been described to suppress intestinal proliferation in weaned or neonatal pigs after a 7-day intervention [21, 22]. One of these studies reports that the supplementation diet decreases expression of β-catenin, the downstream transcription factor in the Wnt pathway [21]. This is in line with our findings that Wnt signaling is increased with cystine restriction.

In conclusion, we show that cystine restriction for two weeks does not seem to induce any systemic effects. However, cystine restriction induces proliferation in the small intestine, and increases the number of goblet cells in the colon. Since more and more people have adopted a vegetarian or vegan diet, it is of importance to study effects of low cystine diets for a longer period of time in order to get more insights in the implications of restriction of dietary intake of sulfur containing amino acids.

## Materials and methods

### Mice

The experiment was approved by the ethics committee of the University Medical Center Utrecht and was in accordance with European law. Eight-week-old male C57BL/6NRJ mice (Janvier) were housed individually in a room with controlled temperature (20–24°C), relative humidity (55% ± 15%), and a 12-h light–dark cycle. Mice were fed and had access to demineralized water ad libitum. Mice (n = 8/group) received either the purified high fat (40 en%), low calcium AIN93-G diet (S9646-E070 Sniff, Germany) or the same diet in which the normally added 0.3% L-cystine was replaced equimolarly by 0.11% alanine (S9646-E072). All mice were acclimatized for 1 week on the AIN93G HF control diet, before the 2-week intervention started.

Body weight was recorded during the intervention. Feces were quantitatively collected during the last 48 hours of the experiment and frozen at −20°C for bile acid measurements. Mice were fasted 4 h before sacrifice. Periorbital puncture or heart puncture were performed to collect plasma after anesthesizing with isoflurane. Mice were sacrificed with cervical dislocation. Gall bladder including its content was collected and centrifuged at 10 000 RCF for 10 min to collect the bile, which was stored at -80 C.

The colon was excised, mesenteric fat was removed, and the colon was opened longitudinally, washed in PBS, and cut into 3 parts. The middle 1.5-cm colon tissue was formalin or carnoys fixed and paraffin embedded for histology. The remaining proximal and distal parts were scraped. Scrapings include the epithelial lining and lamina propria, but not the muscle layer. These scrapings were pooled per mouse, snap-frozen in liquid nitrogen, and stored at −80°C until further analysis. Colonic contents were sampled and snap-frozen for microbiota analysis. A similar procedure was followed for small intestine, which was divided in 4 parts equal in length. Of the last part of the small intestine (ileum), the first/proximal 1 cm was used for histology (swiss roll, formalin fixed), and the remaining part was scraped, snap-frozen in liquid nitrogen, and stored at −80°C until further analysis for mRNA expression.

### qRT-PCR

RNA was reverse transcribed using the iScript cDNA Synthesis Kit (Bio-Rad Laboratories BV, Veenendaal, The Netherlands). Real-time PCR was carried out using FastStart Universal SYBR Green Master Mix (Roche) on a CFX 384 Bio-Rad thermal cycler (Bio-Rad). mRNA expression of genes of interest were normalized to cyclophillin. Primer sequences can be found below (S1 Table).

### Histology and immunohistochemistry

H&E staining was performed to assess the morphology of the tissue. To stain and quantify Ki67-positive cells, paraffin embedded colon sections (5 μm) were deparaffinized and rehydrated in a series of graded alcohols. Sections were incubated for 15 min in 3% H2O2 in PBS to block endogenous peroxidase activity. Sections were placed in antigen retrieval solution (sodium citrate buffer, pH = 6) and heated in a microwave oven for 5 min 700W followed by 20 min 500W, after which they were cooled to room temperature. Sections were blocked with 10% normal goat serum (Sigma-Aldrich Chemie) in PBS-Tween 20 (0.05% v/v) for 30 min. Sections were then incubated with mouse anti-Ki67 (Dako, M724801-8, 1:200) or rabbit anti-Muc2 antibody (NBP1-31231, Novus Biologicals, USA, CO, 1:500) for 1h at room temperature. After washing, slides were incubated with the HRP-conjugated goat anti-mouse (1:200) for 30 min at room temperature. DAB substrate was used for HRP visualization and slides were counter stained with hematoxylin. Ki67 stainings quantification was done by deconvolution of the image in QuPath and measurement of mean intensity in ImageJ, normalized for the haematoxylin signal. 15 crypts per mouse were quantified. Crypt length was measured from the base of the epithelial layer until the bottom of the crypt using ImageJ.

For the High Iron Diamine staining (HID), staining sulfated mucins (brown) and carboxylated mucins (blue), deparaffinized sections were incubated overnight in diamine solution. Then sections were incubated with alcian blue (pH-2.5) for 30 min. Sections were used to measure the thickness of the mucus layer (12 measurements for 3 mice per condition), measured from the surface top to the end of the mucus in the same orientation as the crypts.

### Plasma amino acids measurement

Organic solvents were ULC-MS grade and purchased from Biosolve (Valkenswaard, The Netherlands). Chemicals and standards were analytical grade and purchased from Sigma-Aldrich (Zwijndrecht, The Netherlands). Water was obtained fresh from a Milli Q instrument (Merck Millipore, Amsterdam, The Netherlands). A volume of 15 μL medium was transferred to a labelled 1.5 mL Eppendorf vial. A volume of 285 μL 80% acetonitrile also containing internal standards (final concentration 10 μM) was added and the sample was thoroughly mixed. The sample was centrifuged for 10 min at 17000xg in an Eppendorf centrifuge. A volume of 250 μL supernatant was transferred to a new, labelled Eppendorf vial and evaporated to dryness. Cell samples harvested in 300 μL ice-cold methanol were subjected to the same protocol. The residue was dissolved in 70 μL of borate buffer (pH 8.2) by thorough mixing and the derivatisation was started by adding 20 μL of AccQ-Tag reagent solution prepared according to the suppliers protocol (Waters, Etten-Leur, The Netherlands) after which the samples were vortex mixed and incubated at 55°C for 10 min. The sample was evaporated to dryness and the residue was dissolved in 120 μL 10% acetonitrile containing 0.1 mM formic acid and transferred to an LC sample vial. Analysis was performed on a system consisting of an Ultimate 3000 LC and an Thermo Scientific Q-Exactive FT mass spectrometer equipped with an HESI ion source (Thermo Scientific, Breda, The Netherlands). As a column a Waters HSS T3 (2.1x100 mm, 1.8 μm) was used, kept at a temperature of 40°C in the column oven. Eluent A used for analysis was milliQ water containing 0.1% formic acid, eluent B consisted of acetonitrile containing 0.1% formic acid. The LC gradient used for separation commenced by injecting 1 μL of sample and started at 0% B for 5 min. followed by a 10 min linear gradient to 75% B. In 0.5 min the gradient increase to 100% B and kept there for 1 min before returning to 0% B. The column was allowed to regenerate for 2.5 min prior to a next analysis. Total runtime was 18 min; flow rate was 400 μL/min. The mass spectrometer was operated in ESI-positive mode, full scan 100–1000 m/z, capillary temperature 300°C, sheath gas: 35, aux gas:2, resolution 30000, capillary voltage 3 kV.

### Bile acid measurements

Gall bladder BAs were measured as described in [23]. Fecal BAs were measured as described in [24]). In short, bile samples were diluted with ammonium acetate buffer 15 mM (pH = 8.0): (acetonitrile/methanol = 75/25 v/v) = 50:50, v/v. A mixture of internal standards in methanol was added to the samples to reach a concentration of 2.5 μM. Both fecal and gall bladder BAs were quantified using an Ultra Performance Liquid Chromatography-Mass Spectrometry system (UPLC-MS2, Acquity H-Class Bio UPLC from Waters).

### RNA isolation and sequencing

Total RNA from colon and SI was isolated for qPCR and sequencing, using TRIzol reagent (Invitrogen) according to the manufacturer’s protocol. The RNA was further purified using either RNeasy Minikit columns (Qiagen) or the Nucleospin RNA mini kit (Macherey Nagel). For sequencing, libraries were prepared using Truseq RNA stranded polyA (Illumina) and sequenced on an Illumina Novaseq6000 in paired-end 50 bp reads. Quality control on the sequence reads from the raw FASTQ files was done with FastQC (v0.11.8). TrimGalore (v0.6.5) as used to trim reads based on quality and adapter presence after which FastQC was again used to check the resulting quality. rRNA reads were filtered out using SortMeRNA (v4.3.3) after which the resulting reads were aligned to the reference genome fasta (Mm_GRCm38_gatk_sorted.fasta) using the STAR (v2.7.3a) aligner. Followup QC on the mapped (bam) files was done using Sambamba (v0.7.0), RSeQC (v3.0.1) and PreSeq (v2.0.3). Readcounts were then generated using the Subread FeatureCounts module (v2.0.0) with the Mus_musculus.GRCm38.70.gtf gtf file as annotation, after which normalization was done using the R-package edgeR (v3.28). Differential Expression analysis was performed with an inhouse R-script using DESeq2 (v1.28) taking the raw readcounts as input. Finally a summary report was created using MultiQC (v1.9). Gene set enrichment analysis (GSEA) was performed using the GSEA tool from the Molecular Signature Database (MSigDB), a joint project of UC San Diego and the Broad Institute [25, 26]. Sequencing data has been made available in the NCBI Gene Expression Omnibus (GEO) under accession number GSE234566.

### Fluorescent beads penetration assay

Mucus barrier function was determined in C57BL/6NRJ mice (Janvier) on the low cys (n = 4) or the normal diet (n = 4) (same diets and conditions as described above). Mice were sacrificed and a 1 cm piece of colonic tissue, 2 cm above the rectum, was dissected for mucus measurements. This assay was performed as described in Ijssennagger et al. (2021) [23]. In short, tissue was visualized using a Syto9 green fluorescent nucleic acid stain (ThermoFisher) at the apical side. After 10 mins, FluoSphere Crimson microbeads (1 μm, ThermoFisher) diluted 30 times in Krebs mannitol buffer were added on top of the colon tissue, to measure mucus penetrability. The tissue and beads were visualized using a Zeiss AxioImager Z1 (Carl Zeiss, Germany). Quantifications were done using Fiji and represent the distance of the beads to the cell monolayer.

### Bacterial DNA extraction and 16S sequencing

DNA extraction was performed using a modified protocol of the QIAamp fast DNA stool mini kit (Qiagen, Venlo, the Netherlands) as previously described [27, 28]. In brief, 0.2 g feces was added to ‘lysing matrix A, 2 ml tubes’ (MP biomedicals, Landsmeer, the Netherlands) containing 1 ml InhibitEx buffer (Qiagen). Two rounds of bead incubations were applied at 3.5 m/s for 2 min, followed by 2 min incubation on ice using the FastPrep-24 (MP biomedicals). After 7 min of incubation at 95°C, the protocol of the fast DNA stool mini kit protocol (Qiagen) was followed from the proteinase K treatment step onwards. Total DNA was quantified by Picogreen assay (Thermo Fisher Scientific, Waltham, MA, USA). The 469 bp V3 and V4 hyper-variable regions of the 16S rRNA gene were amplified and sequenced using the Illumina MiSeq instrument and Reagent Kit v3 (600-cycle) according to Fadrosh et al. [29]. Negative controls and mock communities (ZymoBIOMICS microbial community standard (D6300) and ZymoBIOMICS microbial community DNA standard (D6305), ZymoBIOMICS Microbial Community Standard II (Log Distribution) (D6310), Zymo research, USA) were used from the beginning of DNA isolation up to the data analysis stage and matched with the distribution expected mock compositions. For analysis, the QIIME2 microbial community analysis pipeline (version 2021.4) [30] was used with DADA2 for sequence variant detection (with default settings, except for—p-trunc-len-f 255—p-trunc-len-r 240) [31], and SILVA as 16S rRNA reference gene database (SILVA 138) [32]. Sequencing data has been made available on the European Nucleotide Archive under project PRJEB63227.

### Statistical analyses

Statistical tests were performed using Graphpad Prism 9. Mann-Whitney tests or t-tests were performed depending on the distribution of the data as determined using a Shapiro-Wilk test for normality. Separate tests were performed for each gene within the qPCR graphs containing multiple genes using the multiple t-test or Mann-Whitney test function for multiple bars in one graph in Graphpad.

## Supporting information

S1 Fig Dietary cystine restriction is not causing significant damage in the intestine. A) Body weight gain of the mice during the intervention (weight at d14 minus weight at d0 (in gram) (n = 8/group, mean ± SEM). B) Total crypt length (μm) of 15 colonic crypts per mouse (with the median per mouse). C, D) Gene expression of damage markers Ier3, Ripk3 and Birc5 in small intestine (C) and colon (D). (n = 8/group, mean ± SEM).
(TIF)

S2 Fig Significantly differentially expressed genes comparing low cys vs normal cys. A) Heatmap of the 16 significantly differentially expressed genes identified by RNA sequencing on small intestinal scrapings comparing low cys to normal cys (log fold change > 1.5, p-value < 0.05). RNA sequencing on colonic scrapings did not show any significant different genes.
(TIF)

S1 Table Primer sequences. (DOCX)
