# Supplementary figures and images for: Dietary cystine restriction increases the proliferative capacity of the small intestine of mice
Source: PLoS One. 2024 Jan 5;19(1):e0290493. doi: 10.1371/journal.pone.0290493 (PMC10769047; doi:10.1371/journal.pone.0290493)

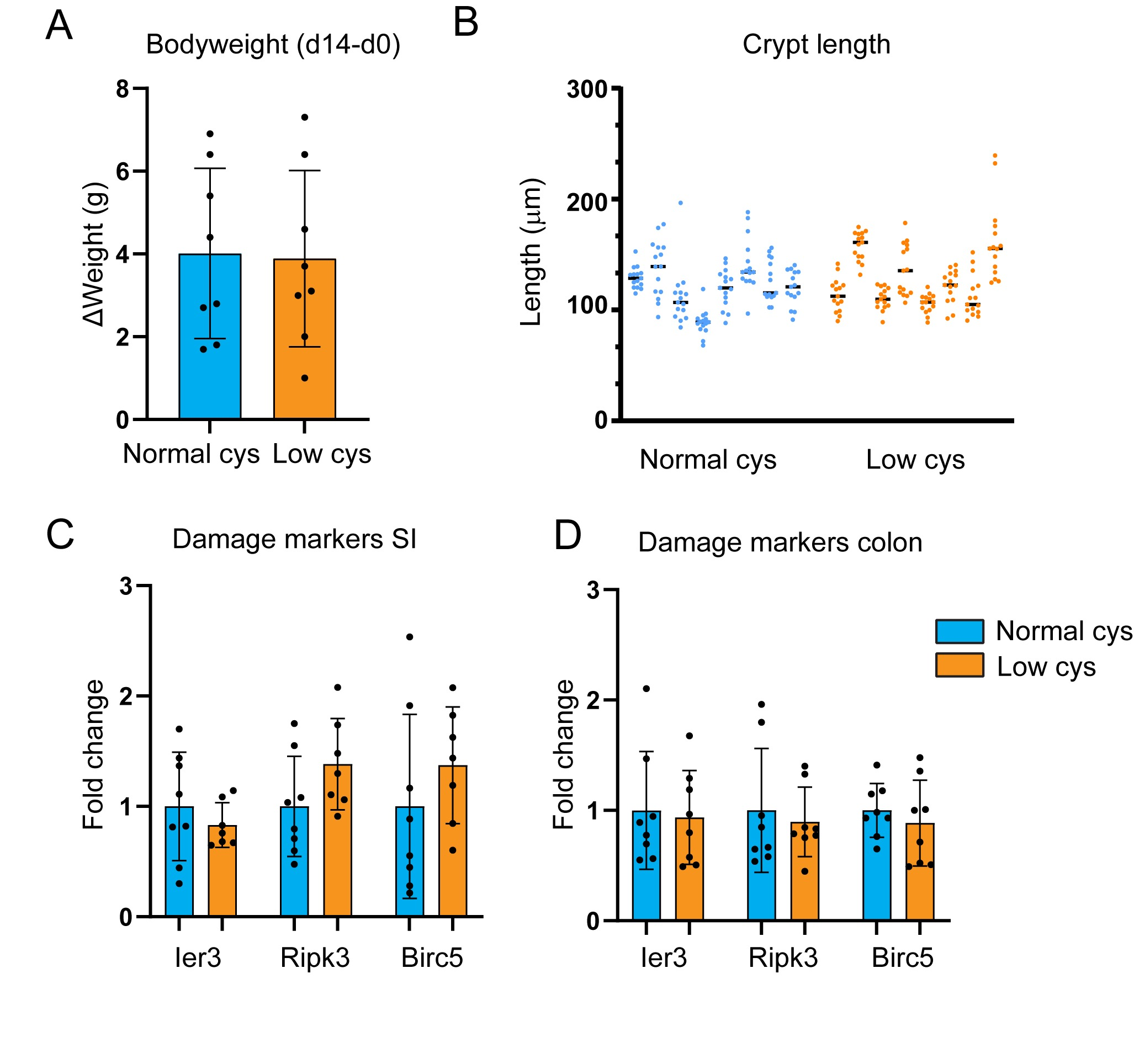

Supplement: S1 Fig — A) Body weight gain of the mice during the intervention (weight at d14 minus weight at d0 (in gram) (n = 8/group, mean ± SEM). B) Total crypt length (μm) of 15 colonic crypts per mouse (with the median per mouse). C, D) Gene expression of damage markers Ier3, Ripk3 and Birc5 in small intestine (C) and colon (D). (n = 8/group, mean ± SEM). (TIF) [file pone.0290493.s001.tif]

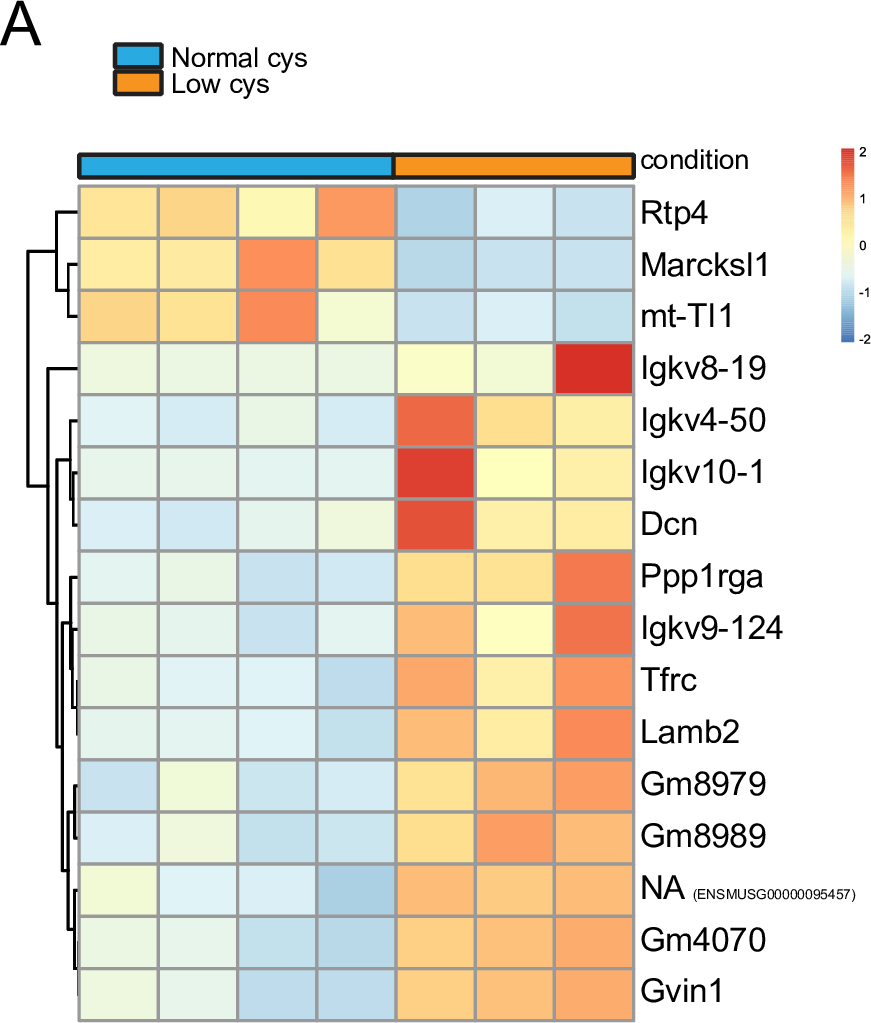

Supplement: S2 Fig — A) Heatmap of the 16 significantly differentially expressed genes identified by RNA sequencing on small intestinal scrapings comparing low cys to normal cys (log fold change > 1.5, p-value < 0.05). RNA sequencing on colonic scrapings did not show any significant different genes. (TIF) [file pone.0290493.s002.tif]
