# Supplementary material for: Dietary cystine restriction increases the proliferative capacity of the small intestine of mice
Source: PLoS One. 2024 Jan 5;19(1):e0290493. doi: 10.1371/journal.pone.0290493 (PMC10769047; doi:10.1371/journal.pone.0290493)
Supplement: S1 Table — (DOCX) [file pone.0290493.s003.docx]

***Table S1: Primer sequences***

| **Gene** | **Forward** | **Reverse** |
| --- | --- | --- |
| Lgr5 | cgggaccttgaagatttcct | gattcggatcagccagctac |
| Sox9 | gaggtttcagatgcagtgagga | tgtcacaacacacgcacaca |
| Olfm4 | tgaaggagatgcaaaaactgg | ctccagcttctctaccaagagg |
| Ki67 | gccataacccgaaagagcag | ccagtttacgctttgcaggt |
| Lyz | cagggtggtgagagatcc | aagcgaggaagtgtgacc |
| Krt20 | tcgaggtccaagtcacggag | gctccagagactctttcatgct |
| Muc2 | tgtggaaccgggaagatg | gaccacaggtatggttctgga |
| Ier3 | tgacacctgagcccatttct | tggtgcctttgtttcttcgg |
| Ripk3 | cgggaaacagtgtgtgacag | ctcggagacagcagcatcta |
| Birc5 | ctcaagaactaccgcatcgc | tcccagccttccaattcctt |
| GSS | acaacgagcgagttgggatg | cctgctttgccagttcttcg |
| GCLC | catcctccagttcctgcaca | tgtactccacctcgtcaccc |
| GCLM | tcagccccgatttagtcagg | tgccatgtcaactgcacttc |
| Axin2 | cattttccgagaacccaccg | gctcagacccctccttttct |
| Ascl2 | actcatgcccacctcgtc | cgtctccaccttactcagct |
| Cmyc | tcagacacggaggaaaacga | cgtctgcttgaatggacagg |
| CyclinD1 | ccccaacaacttcctctcct | ggcttcaatctgttcctggc |
